# Epigenetic-aging-signature to determine age in different tissues

**DOI:** 10.18632/aging.100395

**Published:** 2011-10-26

**Authors:** Carmen M. Koch, Wolfgang Wagner

**Affiliations:** Helmholtz-Institute for Biomedical Engineering; Stem Cell Biology and Cellular Engineering; RWTH Aachen University Medical School, Aachen, Germany

**Keywords:** DNA methylation, epigenetic, predictor, age, tissue

## Abstract

All tissues of the organism are affected by aging. This process is associated with epigenetic modifications such as methylation changes at specific cytosine residues in the DNA (CpG sites). Here, we have identified an Epigenetic-Aging-Signature which is applicable for many tissues to predict donor age. DNA-methylation profiles of various cell types were retrieved from public data depositories - all using the HumanMethylation27 BeadChip platform which represents 27,578 CpG sites. Five datasets from dermis, epidermis, cervical smear, T-cells and monocytes were used for Pavlidis Template Matching to identify 19 CpG sites that are continuously hypermethylated upon aging (R > 0.6; p-value <10^−13^). Four of these CpG sites (associated with the genes NPTX2, TRIM58, GRIA2 and KCNQ1DN) and an additional hypomethylated CpG site (BIRC4BP) were implemented in a model to predict donor age. This Epigenetic-Aging-Signature was tested on a validation group of eight independent datasets corresponding to several cell types from different tissues. Overall, the five CpG sites revealed age-associated DNA-methylation changes in all tissues. The average absolute difference between predicted and real chronological age was about 11 years. This method can be used to predict donor age in various cell preparations - for example in forensic analysis.

## INTRODUCTION

Aging has different consequences in different tissues - it results for example in wrinkle formation of dermis, graying of epidermally-derived hair, loss of bone formation, myeloid bias of blood, and compromised function of the immune system [1]. Despite this wide spectrum of tissue specific age-associated changes the underlying molecular mechanisms might be related. Aging has been associated with accumulation of cellular defects such as DNA damage and telomere shortening. On the other hand, there is accumulating evidence that aging rather resembles a developmentally regulated process which is tightly controlled by specific epigenetic modifications [2-8].

Among epigenetic modifications, DNA methylation is best characterized. CpG dinucleotides in the mammalian genome can be enzymatically methylated at cytosines – and many studies demonstrated the occurrence of age-associated modifications in the DNA- methylation pattern [9-12]. Recently, this research gained further momentum by available technologies such as microarray platforms [13]. Among these the HumanMethylation27 BeadChip facilitates simultaneous analysis of 27,578 CpG sites which are associated with promoter regions of more than 14,000 annotated genes [14]. Previously, we used this microarray for analysis of age-associated DNA methylation changes in mesenchymal stem cells (MSC) and fibroblasts [4, 15, 16]. Despite *in vitro* culture for several weeks these DNA-methylation profiles still reflected age-associated changes that relate to their donors, but this regulation differed markedly between MSC and fibroblasts indicating cell type specificity. Many other authors have used this platform to determine age-associated changes in primary tissues including dermis [17], epidermis [17], blood [11, 18], cord blood [19, 20] and cervical smear [21]. Recently, Bocklandt et al. described a predictor of age for saliva samples which was generated by a dataset of 34 male twin pairs [22]. Based on three CpG sites associated with the genes neuronal pentraxin II (NPTX2), EDAR-associated death domain (EDARADD) and target of myb1 (chicken)-like 1 (TOM1L1) they were able to predict donor age in independent saliva samples [22]. Overall, age-associated DNA-methylation changes are highly reproducible but most of them seem to resemble a tissue-specific phenomenon [12, 23].

On the other hand, some age-associated DNA-methylation changes do not appear to be tissue specific: Teschendorff and co-workers have identified a specific subset of 69 CpGs which are associated with polycomb group protein target genes and which revealed age-associated changes – notably, they described similar modifications in seven independent data sets including normal and cancerous tissues as well as cultured MSC [21]. Furthermore, 10 CpG sites were overlappingly identified upon aging in saliva and blood samples [11, 22]. It is conceivable, that such non-cell type dependent age-associated changes are of central relevance for the underlying process – and they might facilitate age-predictions in heterogeneous cell preparations. Therefore, we have combined several published DNA-methylation datasets to elaborate an Epigenetic-Aging-Signature which can be used for age-predictions across different tissues.

## RESULTS AND DISCUSSION

### Selection of DNA-methylation datasets

**Figure 1:**
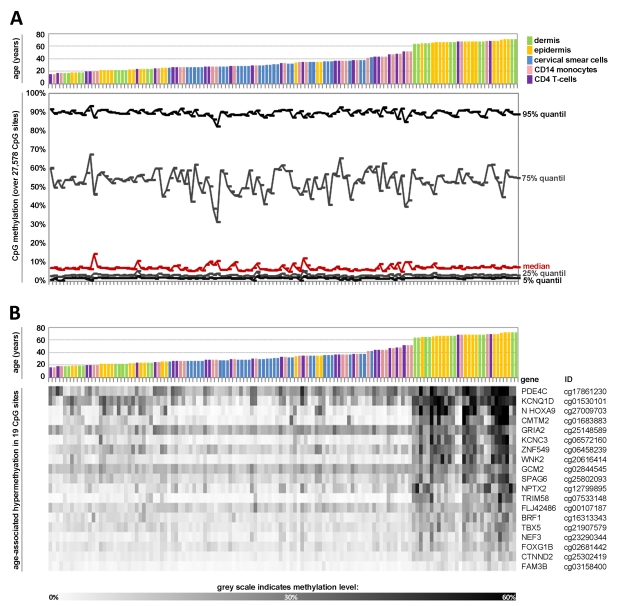
Age-associated DNA-methylation changes at specific CpG sites across different tissues Quantile analysis of beta-values in the 5 datasets of the training-group comprising: dermal cells (predominately fibroblasts), epidermal cells (keratinocytes), cervical smear cells (epithelial cells), and blood (monocytes and T-cells). The global distribution of DNA-methylation did not differ markedly with age or across different tissues (**A**). Pavlidis Template Matching (PTM) identified 19 CpG sites with age-associated hypermethylation (R > 0.6; p-value < 10^−13^) (**B**).

For this study, we have combined several datasets which were retrieved from public data repositories. We have only considered datasets that 1) used the same Infinium HumanMethylation27 BeadChip platform, 2) were generated with freshly isolated cells to exclude effects by culture expansion, 3) used non-cancerous material since malignant transformation might influence age-related changes, and 4) provided reliable information about donor age. DNA-methylation datasets of 13 different cell types or tissues were used: 5 datasets were implemented as a training-set for identification of the Epigenetic-Aging-Signature and 8 datasets were reserved for subsequent validation (table 1). For each of the 27,578 CpG sites the percentage of DNA-methylation was provided as beta value ranging from 0 to 1. Overall, the distribution of DNA-methylation level was similar in all samples of the training-set as determined by quantile analysis of beta-values. There was no clear association between global methylation level and donor age (Figure 1A). Several studies demonstrated that the global DNA-methylation level decreases upon aging [24-26]. However, the HumanMethylation27 BeadChip represents specific CpG sites which are predominantly associated with promotor regions and this might be the reason why global loss of DNA-methylation was not observed.

**Table 1:** DNA-methylation datasets used in this study

Cell type	Tissue	Accession number	Sample number	Age range (median age)	Ref.
**Training group:**					
fibroblasts	dermis	E-MTAB-202	20	18 - 72 yrs (44 yrs)	[17]
keratinocytes	epidermis	E-MTAB-202	30	19 - 72 yrs (47 yrs)	[17]
epithelial cells	cervical smear	GSE20080	30	26 - 43 yrs (32 yrs)	[21]
CD4**^+^** T-cells	peripheral blood	GSE20242	24	16 - 69 yrs (35 yrs)	[11]
CD14^+^ monocytes	peripheral blood	GSE20242	26	16 - 69 yrs (37 yrs)	[11]
**Validation group:**					
leucocytes; buccal epithelial cells	saliva	GSE28746	71	21 - 55 yrs (35 yrs)	[22]
leucocytes	peripheral blood	GSE20236	93	49-74 yrs (63 yrs)	[11]
CD34^+^ HPC	cord blood, peripheral blood	E-MTAB-487	12	0 - 41 yrs (10 yrs)	[19]
lymphocytes	peripheral blood	GSE23638	24	2 - 35 yrs (14 yrs)	[18]
CB MNC	cord blood	GSE27317	168	0 yrs (0 yrs)	[20]
whole blood	peripheral blood	GSE19711	274	52 - 78 yrs (65 yrs)	[21]
breast tissue	breast organoid	GSE31979	15	46 - 68 yrs (53 yrs)	[28]
buccal epithelial cells	saliva	GSE25892	109	15 yrs (15 yrs)	[29]

### Various CpG sites reveal age-associated hyper-methylation

Subsequently, we used Pavlidis Template Matching (PTM) [27] to identify CpG sites which correlated in their methylation level with donor age across the five datasets of the training-group. A template was specified according to the donor age (relative values between 0 and 1) and the beta-values of each CpG site were then compared to this template to identify CpG sites with either continuous hypermethylation or hypomethylation upon aging (Pearson correlation). Initially, we used very stringent parameters with a regression coefficient R of more than 0.6 (corresponding to a p-value <10^−13^). 19 CpG sites passed this criterion - notably, all of them revealed hypermethylation upon aging (Figure 1B). These methylation changes might be influenced by the varying distribution of samples across age groups. To analyze if the 19 CpG sites also revealed age-associated changes within individual datasets we performed PTM analysis for each dataset separately and in most cases this resulted in a similar correlation (Table 2). Subsequently, we used a less stringent cut-off of R > 0.4 (p-value <10^−5^) resulting in age-associated hypermethylation at 431 CpG sites whereas 25 CpG sites were hypomethylated. This is in line with previous reports that demonstrated predominant hypermethylation at specific sites upon aging whereas hypomethylation might be less tightly regulated [11, 21, 22]. Taken together, several CpG sites revealed continuous age-associated methylation changes across all tissue types.

**Table 2:** CpG sites with the most significant age-associated changes

Reference ID	Gene	Age-associated R-values					
all samples of training group	dermis	epidermis	cervical smear	CD14^+^ monocytes	CD4^+^ T-cells
cg06572160	KCNC3	**0.70**	0.73	0.87	0.24	0.41	0.50
cg07533148	TRIM58	**0.69**	0.68	0.76	0.35	0.38	0.52
cg20616414	WNK2	**0.67**	0.66	0.87	0.31	0.32	0.33
cg17861230	PDE4C	**0.67**	0.92	0.91	0.42	0.38	0.22
cg25302419	CTNND2	**0.67**	0.65	0.68	0.33	0.59	0.55
cg25802093	SPAG6	**0.64**	0.63	0.82	0.34	0.31	0.72
cg06458239	ZNF549	**0.63**	0.88	0.85	0.15	−0.13	0.45
cg27009703	HOXA9	**0.63**	0.66	0.90	0.29	0.30	0.56
cg02844545	GCM2	**0.63**	0.75	0.77	0.35	0.66	0.47
cg01683883	CMTM2	**0.63**	0.83	0.80	0.17	−0.18	0.21
cg01530101	KCNQ1DN	**0.63**	0.91	0.67	0.16	0.33	0.45
cg12799895	NPTX2	**0.62**	0.78	0.75	0.04	0.46	0.35
cg21907579	TBX5	**0.62**	0.74	0.73	0.25	0.21	0.35
cg00107187	FLJ42486	**0.62**	0.89	0.65	0.25	0.45	0.44
cg16313343	BRF1	**0.62**	0.79	0.74	0.17	−0.12	0.25
cg25148589	GRIA2	**0.62**	0.90	0.91	0.52	0.25	0.34
cg23290344	NEF3	**0.61**	0.68	0.71	0.26	0.44	0.52
cg02681442	FOXG1B	**0.60**	0.68	0.79	0.08	0.35	0.41
cg03158400	FAM3B	**0.60**	0.71	0.70	0.37	0.13	0.66
cg23571857	BIRC4BP	−0.45^*^	−0.66	−0.84	−0.18	−0.11	−0.40
Pavlidis Template Matching was used to identify CpG sites with the most significant age-associated changes. 19 CpG sites revealed hypermethylation with a Pearson correlation coefficient R of > 0.6 in all samples of the training group. Significant age-associated correlations were also observed in most individual datasets. CpG sites of the Epigenetic-Aging-Signature are indicated in grey. *One additional hypomethylated CpG site (cg23571857) was included in the predictor.							

### Identification of the Epigenetic-Aging-Signature

**Figure 2:**
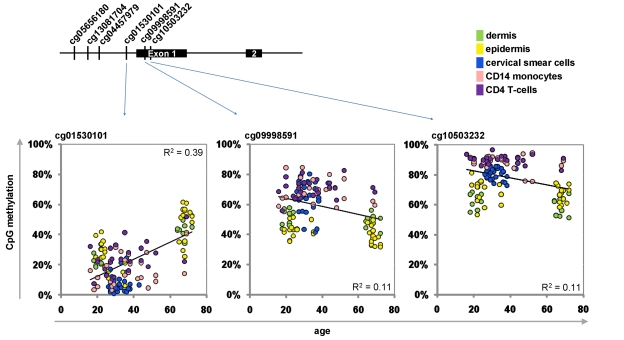
Age-associated hypermethylation and hypomethylation within KCNQ1DN Schematic presentation of the promoter region with six CpG sites represented on the HumanMethylation27 BeadChip. Beta-values of three adjacent CpG sites were plotted against donor age.

Next, we selected a subset of CpG sites to be integrated into the Epigenetic-Aging-Signature. Therefore, we have chosen CpGs which correlated with donor age across the whole training-set as well as in individual datasets. Another criterion was the variation in DNA-methylation level between young and elderly donors as larger changes are less prone to technical noise. Comparison of age-predictions in the training set led us to four hypermethylated CpG sites corresponding to tripartite motif-containing 58 (TRIM58; cg07533148), KCNQ1 downstream neighbor (KCNQ1DN; cg01530101), neuronal pentraxin II (NPTX2; cg1279989) and glutamate receptor ionotropic AMPA 2 (GRIA2; cg25148589). We reasoned that predictions might be more robust by additional consideration of a hypomethylated CpG site. Therefore, we have also included XIAP associated factor-1 (BIRC4BP; cg23571857) despite a lower correlation coefficient (R = -0.45; p = 9.76 x 10^−8^). Selection of CpG sites was irrespective of gene function as it has been shown, that site-specific methylation changes are hardly associated with differential gene expression [4, 15, 18]. Furthermore, we observed age-associated hyper- and hypomethylation in the same promoter region – for example in KCNQ1DN (Figure 2). Notably, the epigenetic age predictor for saliva samples by Bocklandt and co-workers also included the CpG site corresponding to NPTX2 [22] and TRIM58 as well as GRIA2 were also included in their 88 age-related CpG sites. This overlap is remarkable since these authors used different bioinformatic methods and their data was not included in our training-set.

**Figure 3:**
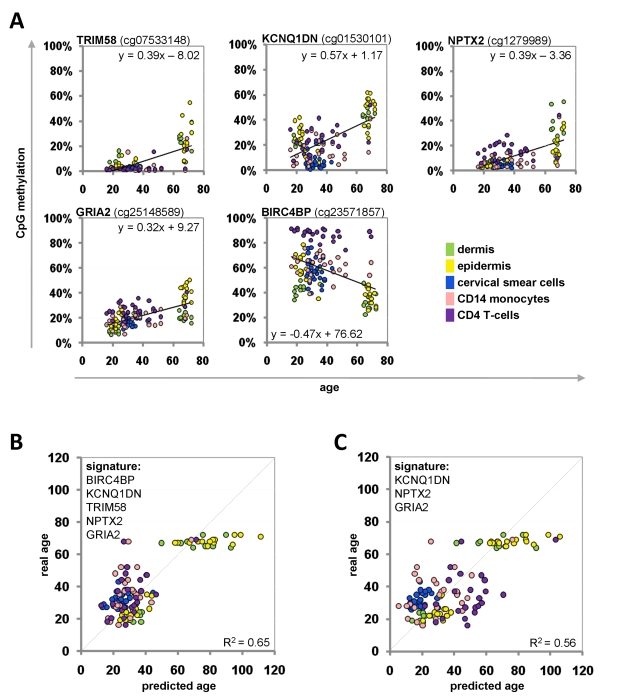
Age-associated DNA-methylation changes at five CpG sites in the training-group Methylation level of five selected CpG sites plotted against donor age. Regression coefficients and equations of linear regression are provided (**A**). Beta-values of the training group samples were used for the linear regression models to predict the donor age (R^2^= 0.65) (**B**). Alternatively, the signature was narrowed down to three CpG sites (R^2^= 0.56) (**C**).

For each CpG site we performed a linear regression analysis: the beta-values were plotted against donor age for all samples of the training-set (Figure 3A). Based on these equations we could inversely calculate donor age for each given beta-value. The mean of the five predictions of the Epigenetic-Aging-Signature was then used to estimate donor age. When we combined all five CpG sites, the predictions correlated with an average precision of ± 9.3 years (Figure 3B). Alternatively, we focused only on three CpG sites with the most significant age-associated correlation (NPTX2, GRIA2 and KCNQ1DN) – even this smaller subset facilitated an average precision of ± 10.3 years in the training-set (Figure 3C).

### Validation of the Epigenetic-Aging-Signature

**Figure 4:**
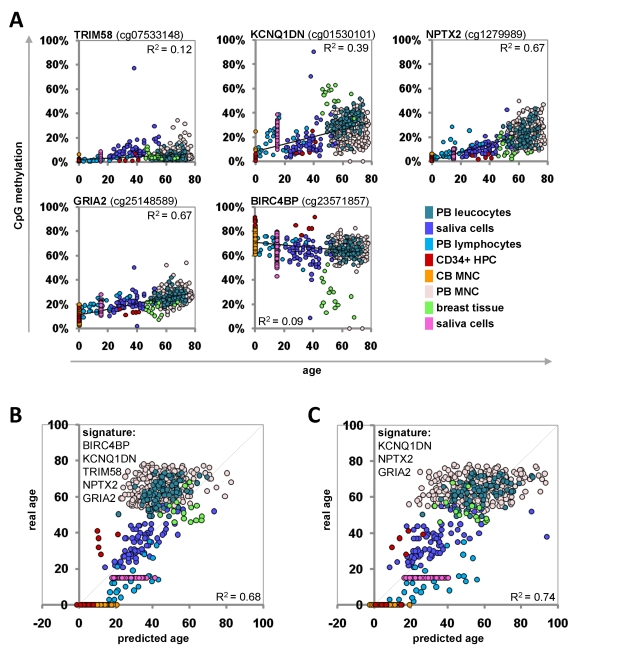
Age-predictions with the Epigenetic-Aging-Signature for the validation group Age predictions were tested with eight independent datasets. Beta-values of the five CpG sites were retrieved and plotted against donor age (**A**). The beta-values were used for the linear regression models of the training-set to predict the age of the donors based on 5 CpG sites (R^2^=0.68) (**B**) or 3 CpG sites (R^2^=0.74) (**C**) of the Epigenetic-Aging-Signature. PB = peripheral blood; CB = cord blood; HPC = hematopoietic progenitor cells; MNC = mononuclear cells.

The Epigenetic-Aging-Signature was then tested on the eight independent datasets of the validation-group (Table 1). To this end, we have only considered the five beta-values which corresponded to the CpG sites of the Epigenetic-Aging-Signature. Each of these CpG sites revealed age-associated changes in analogy to the training-set (Figure 4A). The beta-values were then used for the linear-regression models of the training-group to estimate the donor age. The predictions for donor age in the validation-group also correlated with the real age with an average precision of ± 12.7 years (Figure 4B). These predictions were even improved when we focused on the three most significant CpG sites of the signature (KCNQ1DN, NPTX2 and GRIA2) – then the average precision was ± 11.4 years (Figure 4C). For some individual datasets the precision was even less than 6 years. Gender-related differences in the age-predictions were not observed using this signature (data not shown).

Epigenetic changes are a hallmark of aging - but it is yet unclear how these modifications are regulated [6]. DNA-methylation changes have been shown to be enriched in target genes of polycomb complexes [21] or bivalently modified DNA [11]. Recently, we have demonstrated that long-term culture related DNA-methylation changes in MSC are associated with repressive histone marks [2]. Thus, it may be speculated that protein complexes which are associated with the histone code are involved in this process.

## CONCLUSION

In this study we have identified an Epigenetic-Aging-Signature consisting of five CpG sites which facilitates predictions of donor age across different tissue types. This method can for example be used in forensic analysis to estimate donor age of unknown tissue specimen including blood. It has to be noted, that chronological age is not identical with biological age and it is conceivable that some of the discrepancy between predicted and real age can be attributed to this difference – further research might facilitate determination of the biological age for personalized medicine.

## METHODS

### DNA-methylation profiles used in this study

In this study we have considered all at the time publically available datasets with the Infinium HumanMethylation27 BeadChip platform in the public repositories Gene Omnibus (http://www.ncbi.nlm.nih.gov/geo; GPL 8490) and Array Express (http://www.ebi.ac.uk/arrayexpress; A-GEOD-8490). After literature search we decided to include the following 13 datasets for subsequent analysis which were divided in a training-group for identification of the Epigenetic-Aging-Signature and a validation-group.

The authors of these important primary studies have to be acknowledged: Grönniger and co-workers isolated keratinocytes from epidermal suction blisters and dermal fibroblasts from punch biopsies (E-MTAB-202) [17]. Epithelial cells from cervical smear samples (19 HPV negative controls, 11 HPV positive controls) were collected and analysed as described by Teschendorff et al. (GSE20080) [21]. Leucocytes, CD4^+^ T-cells and CD14^+^ monocytes were isolated from fresh venous whole blood as described by Rakyan and co-workers (GSE20242 and GSE20236) [11]. Saliva samples comprising buccal epithelial cells and leucocytes were collected as described in detail by Bocklandt et al. (GSE28746) [22]. CD34^+^ hematopoietic progenitor cells (HPC) were isolated from cord blood and from G-CSF mobilized peripheral blood as described by Bocker and colleagues [19] (E-MTAB-487; monocytes and granulocytes were not included to keep the cell specification homogeneous). Peripheral blood lymphocytes were isolated from whole blood as described in Chen et al. (GSE23638) [18]. Mononuclear cells were harvested by centrifugation of whole blood isolated from umbilical cord blood (GSE27317) [20]. Teschendorff and co-workers analyzed whole blood samples of healthy postmenopausal women (GSE19711) [21]. Normal breast organoids prepared by enzymatic digestion of reduction mammoplasty specimens were analyzed by Fackler et al. (GSE31979) [28]. Essex and colleagues determined DNA-methylation profiles in saliva samples of fifteen-years-old adolescents (GSE25892) [29]. Age ranges and sample numbers are summarized in Table 1.

### Combination of different datasets

Beta-values of the different datasets were combined by the reference ID of the Infinium HumanMethylation27 BeadChip platform (Illumina Inc., San Diego, CA, USA). These beta-values represent the percentage of methylation at each of the 27,578 CpG sites – they are continuous variables between 0 and 1 and represent the intensity ratio of the methylated bead to the combined locus intensity. Background normalized raw data of these beta-values were determined with the BeadStudio software (Illumina) and retrieved from the public data repositories Gene Onmibus and Array Express. Initially we considered various normalization regimen including quantile normalization to minimize chip effects [30]. On the other hand, it is expected that methylation patterns vary between different cell tissues and this would be masked by such normalization regimen. Beta-values are less affected by normalization than the relative gene expression changes in mRNA microarray data. Furthermore, non-normalized beta-values are usually in line with validation experiments by pyrosequencing [4, 14, 15]. Therefore, we decided to use non-normalized raw-data for comparison over all data-sets. The combined data table of the training-set was subsequently analyzed using the MultiExperiment Viewer (MeV6.2) [31].

### Identification of the Epigenetic-Aging-Signature

To identify CpG sites which reveal continuous age-associated hypermethylation or hypomethylation we performed Pavlidis Template Matching (PTM) [27] with the MultiExperiment Viewer (MeV6.2) [31]. Each sample of the training-set was matched to a template with corresponding donor age. The combined dataset was then searched for CpG sites which correlated linearly in their beta-values with the donor age of the template (Pearson correlation) – initially we used very stringent criteria with R > 0.6. In analogy, each dataset was analysed separately and the overlap of age-associated changes supported the notion that they occur in different tissues. Based on this analysis, we selected five CpG sites which revealed the best age-associated correlation across all 5 datasets of the training-set and relevant variation in the beta-values. For simplicity they were termed by their corresponding genes: TRIM58 (cg07533148), KCNQ1DN (cg01530101), NPTX2 (cg1279989), GRIA2 (cg25148589) and BIRC4BP (cg23571857).

For each of these CpG sites we performed a linear regression analysis of beta-values *versus* donor age with EXCEL 2007 (Microsoft). These linear regression models were then used for age-predictions in the datasets of the training-group as well as for the validation-group: the five CpG sites (*i*) were inversely used to predict the age (*N*) by inserting the specific DNA-methylation levels of the corresponding CpG site (*β*).

$$N_{i}=(\beta_{i}-A_{i})/B_{i}$$

Where *A* is the Y-axis intercept and *B* is the slope of the corresponding CpG site in the training group (Figure 3A). The mean of the predictions of the five individual CpG sites of the Epigenetic-Aging-Signature was subsequently used to predict donor age.
